# Phase-Type Fresnel Zone Plate with Multi-Wavelength Imaging Embedded in Fluoroaluminate Glass Fabricated via Ultraviolet Femtosecond Laser Lithography

**DOI:** 10.3390/mi12111362

**Published:** 2021-11-04

**Authors:** Qisong Li, Xuran Dai, Haosong Shi, Yi Liu, Long Zhang

**Affiliations:** 1 Shanghai Key Lab of Modern Optical System, University of Shanghai for Science and Technology, Shanghai 200093, China; liqisong@usst.edu.cn (Q.L.); XURAN001@e.ntu.edu.sg (X.D.); shihaosong123@163.com (H.S.); 2 Key Laboratory of Materials for High Power Laser, Shanghai Institute of Optics and Fine Mechanics, Chinese Academy of Sciences, Shanghai 201800, China; 3 Division of Physics and Applied Physics, School of Physical and Mathematical Sciences, Nanyang Technological University, Singapore 637371, Singapore; 4 CAS Center for Excellence in Ultra-Intense Laser Science, Shanghai 201800, China

**Keywords:** ultraviolet femtosecond laser fabrication, inner refractive index modification, phase-type Fresnel zone plate

## Abstract

Herein, we report a novel optical glass material, fluoroaluminate (AlF_3_) glass, with excellent optical transmittance from ultraviolet to infrared wavelength ranges, which provides more options for application in optical devices. Based on its performance, the phase-type Fresnel zone plate (FZP) by ultraviolet femtosecond (fs) laser-inscribed lithography is achieved, which induces the refractive index change by fs-laser tailoring. The realization of ultraviolet fs-laser fabrication inside glass can benefit from the excellent optical performance of the AlF_3_ glass. Compared with traditional surface-etching micro-optical elements, the phase-type FZP based on AlF_3_ glass exhibits a clear and well-defined geometry and presents perfect environmental suitability without surface roughness problems. Additionally, optical focusing and multi-wavelength imaging can be easily obtained. Phase-type FZP embedded in AlF_3_ glass has great potential applications in the imaging and focusing in glass-integrated photonics, especially for the ultraviolet wavelength range.

## 1. Introduction

Fresnel zone plates (FZPs) are binary planar micro-optical elements consisting of a series of radially symmetric rings that can tailor the amplitude and phase of light [1,2]. This focusing mode can effectively reduce spherical aberration in comparison with refractive microlenses. In addition, because of their planar structure and low volume, FZPs can be easily incorporated into miniaturized and integrated optical systems. Owing to their attractive characteristics, FZPs have been widely used in various fields, including X-ray microscopy [3], optical trapping [4,5], pulse shaping [6], optical interconnections [7], optical imaging [8], and THz focusing [9]. To date, many methods have been developed to fabricate FZPs, such as photolithography [10], ion-beam and e-beam lithography [3,11], and femtosecond laser direct writing (FsLDW) [12,13,14,15]. However, most of these methods emphasize the use of photosensitive materials, which are generally disregarded in technical applications because of their fragile and unstable nature. The development of excellent optical dielectric materials and fabrication of FZPs based on optical materials remains one of the most significant challenges for the application of optical devices. For the fabrication of optical dielectric materials, femtosecond laser nonlinear lithography can be considered a favorable manufacturing technology to ensure precise dielectric material processing because a laser with ultrashort pulse duration can suppress the heat-affected zone and generation of debris around the damaged crater [16,17], achieving high-precision microstructure fabrication. Usually, femtosecond laser nonlinear lithography is realized in two ways: surface ablation [18,19,20,21] and inner waveguide fabrication [22,23,24]. However, femtosecond laser surface ablation results in relatively large surface roughness and low optical performance. Post-processing technologies (for example, chemical etching, dry etching, and laser annealing) are imperative for surface fabrication [25,26,27,28]. The representative fabrication method of femtosecond laser lithography is proposed by Soh et al. [28]. The FZP first was fabricated on the UV photoresist by femtosecond laser direct fabrication, and then the structure was transferred onto the fused silica by the buffered oxide etch. Therefore, inner waveguide fabrication using a femtosecond laser may be a suitable candidate and requires consideration. The FZP inside Sapphire by femtosecond laser inner waveguide fabrication has also been reported [29].

However, the performance of optical dielectric materials is critical for the application of optical devices. In terms of optical dielectric materials, sapphire is the competitive candidate from the ultraviolet to the mid-infrared spectral region, but is expensive and has weak performance at high temperatures. Silicate glasses are the most common material, but they only work at visible wavelengths. Fluoride glass can be regarded as one of the most appropriate candidates for optical devices from the ultraviolet to the mid-infrared spectral region and can work under high temperatures. This can be attributed to the low intrinsic loss and distinctive glass formation system of fluoride glass. In our previous work [30,31], oxyfluorogallate glass (FGa glass) was reported to achieve excellent optical performance in the infrared wavelength range as well as structural damage characteristics resulting from fabrication using a femtosecond laser. However, considering the ultraviolet region, fluoroaluminate glass is better for fabricating ultraviolet optical devices because of its higher transmissivity compared with that of FGa glass. The microstructure fabrication on the fluoroaluminate glass induced by femtosecond laser has not been studied until now. This paper proposes a promising approach for the fabrication of fluoroaluminate glass via femtosecond-laser-inducing multiphoton absorption. This internal refractive index results change, which offers compelling advantages. The refractive index of the designated region can be tailored without any damage to the mechanical properties of the material.

We first investigated the optical transmission properties of the fluoroaluminate glass material and calculated the bandgap from the ultraviolet cutoff wavelength. The mechanism of action resulting from fabrication using a femtosecond laser was suggested based on the laser wavelength and material bandgap. Then, a phase-type FZP based on fluoroaluminate glass was fabricated via ultraviolet femtosecond laser-inscribed lithography by changing the refractive index. This type of optical device exhibits a clear and well-defined geometry in optical microscopy and is devoid of surface roughness problems, unlike traditional surface-etching micro-optical elements. Moreover, optical focusing and multi-wavelength imaging are easily achieved using the phase-type FZP. In addition, a software simulation was qualitatively performed to analyze the focusing ability of the FZP and the multi-wavelength imaging results.

## 2. Materials and Methods

Glass samples with a major molar composition of 35AlF_3_-15YF_3_-20CaF_2_-10MgF_2_-10SrF_2_-10BaF_2_, referred to as AlF_3_ glass, were prepared via conventional melting and casting at 1000 °C for 120 min in a covered platinum crucible under a nitrogen atmosphere. After sufficient melting, the glass melt was cast by being poured into a preheated platinum mold and was annealed in a furnace near the glass transition temperature. The experimental samples were cut to a diameter of 18 mm and a thickness of 1 mm and then polished for optical property measurements. The morphology of each sample was observed using optical microscopy and field emission scanning electron microscopy (FE-SEM, ZEISS, Jena, Germany) with energy dispersive spectroscopy (EDS). The optical absorption and transmission spectra of the samples were recorded in the range of 250–3000 nm using a Perkin–Elmer Lambda 750 UV/VIS/NIR spectrophotometer.

**Figure 1:**
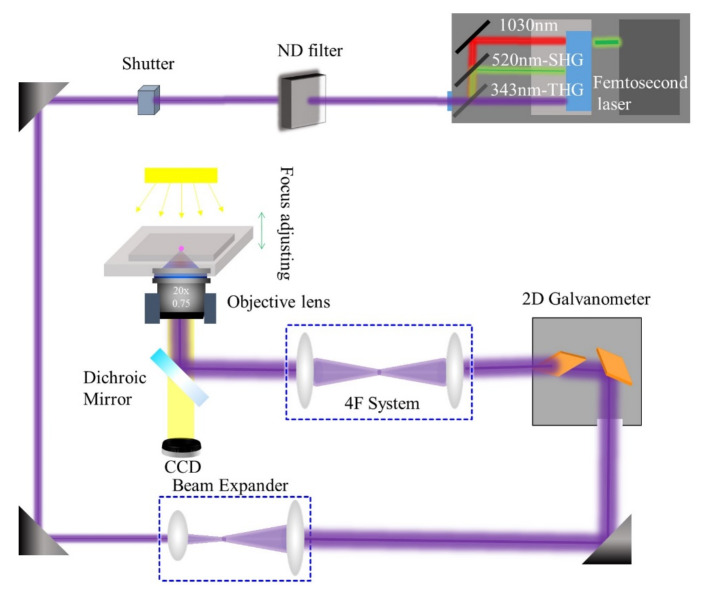
Schematic of experimental setup for the ultraviolet femtosecond laser fabrication.

A diagram of the FsLDW micro/nanofabrication system is shown in Figure 1. The fabrication of these samples was performed under normal atmospheric conditions using an ultrafast amplifier with direct diode-pumped technology. A central wavelength was generated at 1040 nm; two other wavelengths were generated at 520 and 343 nm, with second- and third-harmonic generation with a high repetition rate of 200 kHz. The laser wavelength *λ* used in the experiment was 343 nm, the pulse duration was 220 fs, and the repetition rate was 200 kHz. The objective was from Olympus with a multiple of 20× and an N.A. of 0.75. The output laser beam was first expanded and then entered the galvanometer system. Using the 4F lens system, the laser-focusing spot can be projected onto the objective. By designing the required structure, the data coordinates of the structure can be imported into the system-controlling software, thereby enabling 3D fabrication. In our experiments, all measurements were conducted at room temperature (~25 °C).

## 3. Results and Discussion

**Figure 2:**
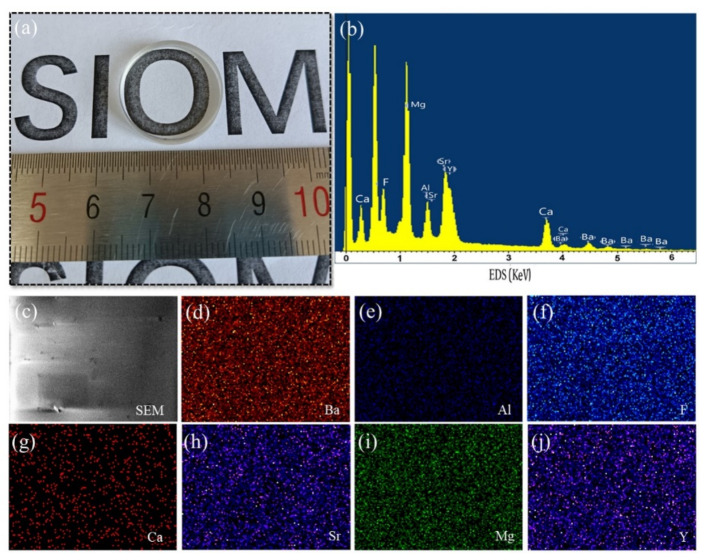
(**a**) Optical picture of the AlF_3_ glass and (**b**–**j**) EDS result and the map scanning mode of AlF_3_ glass for chemical elements stated.

Generally, a wide range of ions in glass materials play a key role in the optical performance of the glass composition, such as optical transmissivity. The optical image created by the AlF_3_ glass with a ~5 mm thickness (Figure 2a) exhibits excellent visible transmissivity with respect to human eyes, and the letters “SIOM” can be clearly observed through the AlF_3_ glass. To determine the ion composition, SEM-EDS was used to analyze the elemental details; the results are shown in Figure 2b–j. Figure 2b presents the entire EDS spectrum, in which the negative ion is the F ion, and the positive ions are Al, Y, Ca, Mg, Sr, and Ba. The map-scanning results for the corresponding ions are displayed in Figure 2d–j, and the corresponding SEM image is shown in Figure 2c. It clearly reveals the ion components of the AlF_3_ glass. For optical performance, the ion excitation energy is a critical evaluation factor. Theoretically, the formula for the excitation energy can be expressed as

(1) hv=E+M−φ

where *h* is Planck’s constant, *v* is the photon frequency, *E* is the electrophilic potential of the anion, *M* is the work done by overcoming the Coulomb action of anions and cations, and *φ* is the energy gained by the anions through polarization. In this glass system, the F ion possesses a large electric potential, requiring a large amount of energy to be excited. The high valence state of the positive ions generates a large ion radius, which results in a larger excitation energy. As a result, AlF_3_ glass exhibits a very short UV absorption cutoff wavelength and excellent UV optical performance.

**Figure 3:**
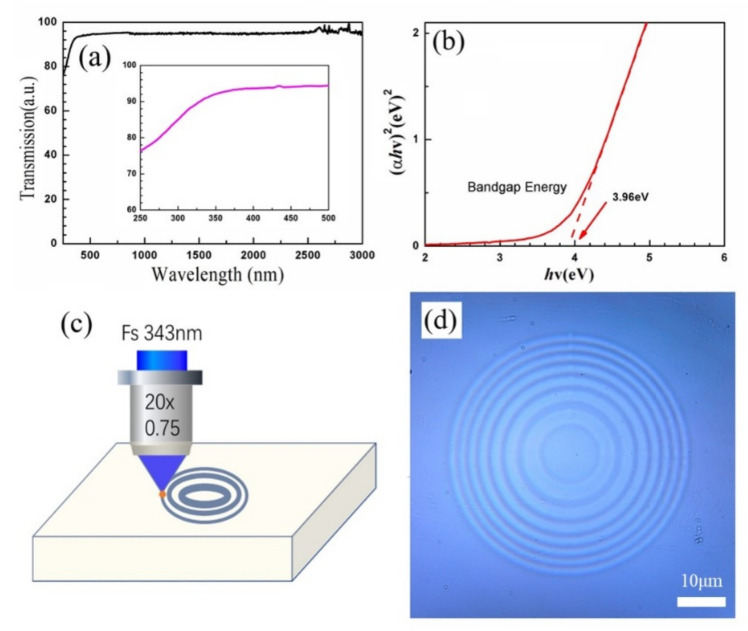
(**a**) Optical transmissivity and (**b**) bandgap of AlF_3_ glass. The inset shows details of the ultraviolet transmissivity. (**c**) Fabrication diagram and (**d**) optical picture for the fabricated Fresnel zone plate inside the AlF_3_ glass.

To investigate the optical performance for potential applications in optical window materials, the UV/VIS/NIR transmission spectra for a sample thickness of ~1 mm were measured. The results are presented in Figure 3a. The AlF_3_ glass demonstrated an excellent near-infrared and visible transmittance of ~92% from 400 nm to 3 μm. The transmission loss was primarily attributed to the surface Fresnel reflections and the scattering due to the sudden change in the refractive index associated with the interference between the air and the glass surface. The intrinsic absorption of the AlF_3_ glass material can be ignored, and no impurities are produced during the preparation process. More importantly, the UV transmittance of AlF_3_ glass can be approximately 76% at a wavelength of 250 nm, as indicated in the inset of Figure 3a. Therefore, AlF_3_ glass is a suitable candidate for full-wave band optical applications, particularly in the ultraviolet wavelength range. The excellent optical transmissivity of this glass also provides the possibility of ultraviolet femtosecond laser fabrication. The absorption edge of the glass material is attributed to the bandgap (*Eg*), which can be calculated from the Urbach plot expressed as an (α*hν*)^2^–*hν* diagram, using the Formula (2) [32]:

(2) $$\left\{\begin{array}{c} \alpha =\frac{1}{d}\ln\frac{1}{T}\\ \alpha hv=B{\left(hv-E_{g}\right)}^{1/2} \end{array}\right.$$

where *α* is the linear absorption coefficient, *d* is the sample thickness, *T* is the transmissivity, and *hυ* and *B* represent the photon energy and a constant, respectively. The value of the virtual bandgap Eg can be extracted from the intercept of the tangent of the Urbach plot curves, which are plotted as the calculated (*αhν*)^2^ versus *hν*, as illustrated in the inset of Figure 3b. From this curve, the bandgap energy of AlF_3_ glass can be calculated as ~3.96 eV, corresponding to a wavelength of 313 nm (*λ* = 1240/Eg).

To leverage the excellent optical properties of AlF_3_ glass, we fabricated a phase-type FZP inside the glass using ultraviolet femtosecond-laser-inscribed lithography. Considering that the laser wavelength (343 nm) is larger than the bandgap of AlF_3_ glass (313 nm), two photons are required to excite an electron from the valence band to the conduction band. Thus, the nonlinear action mechanism for the femtosecond laser interaction with the AlF_3_ glass can be regarded as a two-photon absorption effect, which results in a change in the refractive index. Next, we designed the FZP with the outer radius *r_m_* of the *m*th zone using the following equation [25,33]:

(3) $$r_{m}^{2}+f^{2}=\left(f+m\lambda /2\right)\;f\approx \frac{r_{m}^{2}}{m\lambda }\;m=1,2,\ldots ,N$$

where *f*, *λ*, and *r_m_* are the focus, wavelength, and outer radius of the *m*th zone, respectively. By controlling the focal spot inside the glass and moving along the designed path, the phase-type FZP could be fabricated by changing the refractive index via the fs-laser two-photon absorption effect. As an example, an FZP with a wavelength of 808 nm, a focus of 80 μm, and inner and outermost diameters of ~14 and ~53 μm, respectively, was adopted in our experiment. The processing laser power and scan step were optimized to be approximately 42 mW and 100 nm, respectively. A fabrication diagram of the FZP inside the glass is presented in Figure 3c. As shown in Figure 3d, the optical image provided by the phase-type FZP displays a clear profile of circular rings and a high optical contrast difference by changing the refractive index without any material damage. However, it is difficult to determine the magnitude of the change in refractive index from the femtosecond laser two-photon absorption owing to the microscale structure inside the glass. The optical performance can also be qualitatively evaluated by measuring the focusing and imaging properties.

**Figure 4:**
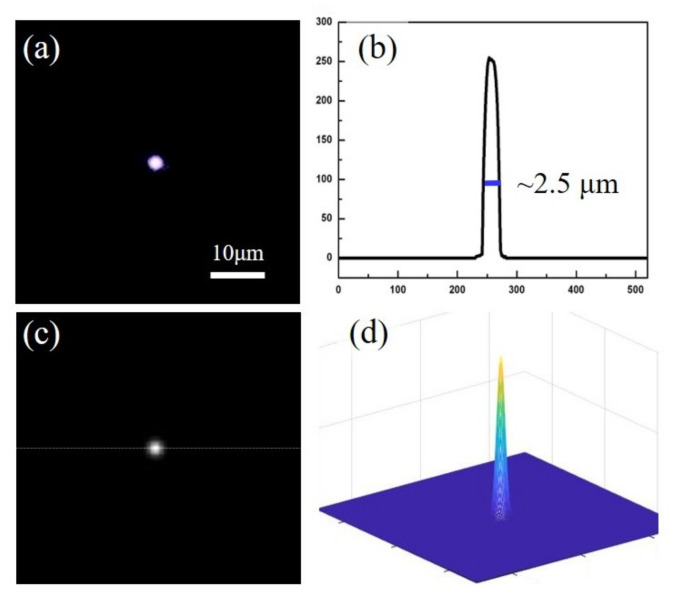
(**a**) Simulation and (**b**) experimental results for the focusing performance of the Fresnel zone plate under an 808 nm laser. (**c**,**d**) Corresponding sectional views of the focus intensity.

Figure 4 shows the experimental and simulation results for the FZP in terms of the focusing performance. A semiconductor laser with a wavelength of 808 nm was used as the irradiation source. The focusing result in the transmission direction was measured at a position of ~81 μm, as depicted in Figure 4a. This was approximately consistent with the designed focal length. The cross-profile distribution of the optical intensity is displayed in Figure 4b. The width of the focus was approximately 2.5 μm, determined by measuring the beam size at the 1/*e* position of the full width of the beam. The ratio between the focus and image size was approximately 0.048. Furthermore, the FZP was simulated using MATLAB to verify the focusing performance and analyze the refractive index change. In the case of the diffraction of light via an FZP, the distribution of the optical field can be calculated according to Kirchoff’s diffraction formula:

(4) $$u\left(\xi ,\zeta \right)=\frac{\exp\left({\mathrm{ik}z}_{d}\right)}{{i\lambda z}_{d}}\int_{-\infty }^{\infty }\int_{-\infty }^{\infty }g\left(x,y\right)\exp\left\{\frac{\mathrm{ik}}{2z_{d}}[\left({\left(\xi -x\right)}^{2}+{\left(\zeta -y\right)}^{2}\right)]\right\}\mathrm{dxdy}$$

$$g\left(x,y\right)=\left\{\begin{array}{c} \;\exp\left(i\pi \Delta n*d_{p}/\lambda \right)\;\mathrm{odd}\\ \;1\;\mathrm{even} \end{array}\right.$$

where *g*(*x*,*y*) is the complex amplitude distribution of the diffraction screen, which is determined by the FZP; *z_d_* is the distance between the FZP and the observation surface; *dp* and ∆*n* are the thickness and refractive index changes of the FZP, respectively; and (*ξ*,*ζ*) is the coordinate of the observation surface. Then, the optical field distribution can be calculated using the Fourier transform for the observation surface:

(5) $$\left\{\begin{array}{c} F=fftshift\left(fft2\left(u\left(\xi ,\zeta \right)\right)\right)\\ I=F.*\mathrm{CONJ}\left(F\right) \end{array}\right.$$

Subsequently, we simulated the focus intensity under irradiation by an 808 nm semiconductor laser, as illustrated in Figure 4a. In the simulation, we assumed that the thickness of the FZP was approximately 10 μm and that ∆*n* was designed to be 0.01, which is the most common value of the refractive index change induced by a femtosecond laser [34]. The theoretical focusing performance of the FZP was confirmed, as shown in Figure 4c,d. We divided the observation screen for 400 px, and the focusing size was obtained at the 1/*e* position of the beam full width at the transmission position *z* = 60 μm. Then, we measured the ratio between the theoretical focusing size and screen size to be approximately 0.04. However, the focal position was not very accurate due to the microscale structure inside the glass, which produced an error between the measurement and the theoretical value. Nevertheless, this simulation qualitatively demonstrated the focusing ability of the phase-type FZP. The exact measurement of the refractive index changes of the inner waveguide induced by the femtosecond laser should be performed according to the diffraction method [29].

**Figure 5:**
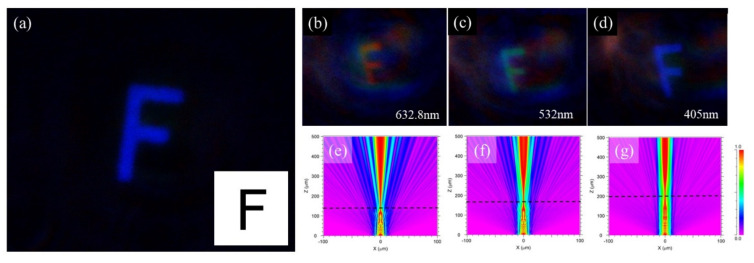
(**a**) Imaging results with a white light source. (**b**–**d**) Imaging results with red (632.8 nm), green (532 nm), and blue (405 nm) light sources. (**e**–**g**) Simulated imaging paths for the red, green, and blue lights.

**Figure 6:**
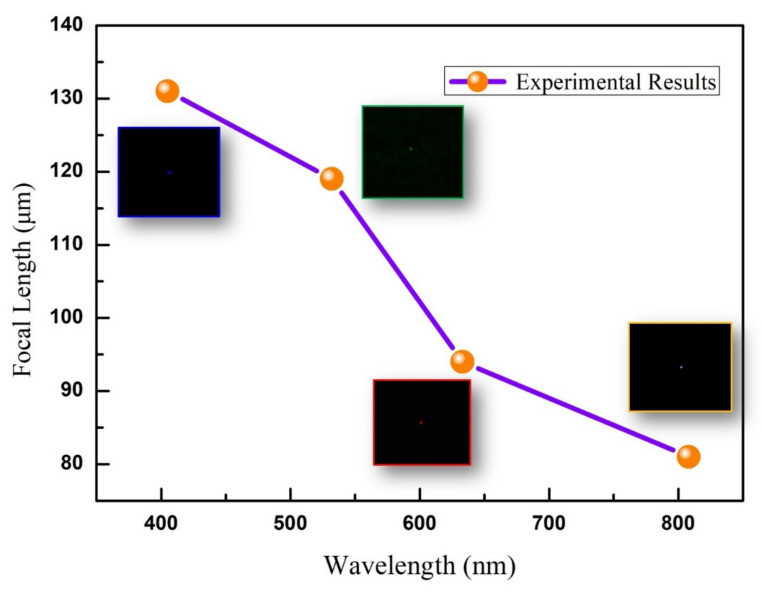
Experimental results for the focal length vs. different wavelengths (808 nm, 632 nm, 532 nm, and 405 nm), the insets are the pictures of the focal point.

Another important feature of the FZP is its imaging characteristics. The imaging performance of the developed FZP is presented in Figure 5a–d. Figure 5a shows an image of the letter F when white light is irradiated. It shows very clear imaging results, and the main color is blue because the CCD is more sensitive to blue light. To investigate the multi-wavelength imaging features, the lasers with different wavelengths were used as the light sources: red (633 nm), green (532 nm), and blue (405 nm). The multi-wavelength imaging results are shown in Figure 5b–d. It can be seen that this phase-type FZP has an acceptable imaging ability for red (633 nm), green (532 nm), and blue (405 nm) light. Moreover, the clear imaging positions display the increasing tendency as the wavelength increases. We then simulated the spreading light path of the FZP for different wavelengths, as shown in Figure 5e–g. The distribution of the optical field exhibits focusing paths for red (633 nm), green (532 nm), and blue (405 nm) lights. According to the light paths, a shorter wavelength of incident monochromatic light corresponds to a longer focal length, which is consistent with the equation for the FZP. Furthermore, we also measure the focal lengths of phase-type FZP for different wavelengths, which is shown in Figure 6. From the measuring results, the focus positions also display an increasing tendency as the wavelength increases and the dependence is approximately linear. Both the simulation and experimental results agree with the variation tendency observed in the imaging experiments (Figure 5b–d). Therefore, the imaging distance is directly proportional to the wavelength of the diffractive optical element, whereas it is inversely proportional to the refractive lens. The FZP devices with different materials and fabrication methods are compared in Table 1. From the comparison, it can be seen that the FZP inside AlF_3_ glass by femtosecond-laser-induced refractive index change technology shows acceptable focusing and imaging properties. Due to the multi-level structure in Sapphire by the same fabrication method, the FWHM is better than that in this work. This relation can be used to eliminate chromatic aberration by combining diffractive and refractive optical elements. However, we also find that there exists some deviation for the focal lengths between the measurement in Figure 6 and the simulation in Figure 5e–g. This is because the refractive index change is not very accurate. We will try to get an accurate method to measure the refractive index change in this work and increase the focal performance in the next step.

## 4. Conclusions

In summary, we reported a new optical material, called AlF_3_ glass, which displays excellent optical performance in the ultraviolet to near-infrared wavelength range and is thus highly suitable as an optical device. We achieve the device fabrication of a phase-type FZP embedded in the AlF_3_ glass using an ultraviolet femtosecond laser based on the two-photon absorption effect. The fabricated phase-type FZP exhibited a clear and well-defined geometry. Notably, it is devoid of the surface roughness problem associated with laser surface processing and presents perfect environmental suitability. More importantly, optical focusing and multi-wavelength imaging were achieved by the phase-type FZP, which was confirmed through the simulation. This novel glass and microstructures with such merits can be used in practical applications requiring exceptional ultraviolet micro-optics, such as hybrid diffractive/refractive achromatic systems.

## Figures and Tables

**Table 1:** Comparison of the experimental results for some selected FZP devices with different materials and fabrication methods.

Fabrication Methods	Materials	FWHM	Imaging	Reference
Femtosecond laser writing and chemical etching	Sapphire	1.85 μm	Single wavelength	[25]
Laser-induced solid ablation	Metal-coated borosilicate glass	5.95 μm	Multi-wavelength	[21]
Femtosecond laser focal field engineering	Polymer	None	White light	[15]
Femtosecond laser lithography and chemical etching	Fused silica glass	2.02 μm	Single wavelength	[28]
Femtosecond-laser-induced refractive index change	Sapphire	1.75 μm with four-level FZP	Multi-wavelength	[29]
Femtosecond-laser-induced refractive index change	Fluoroaluminate glass	2.5 μm with second-level FZP	Multi-wavelength	This work

## Data Availability

Not applicable.
